# Cross-tissue molecular responses in the liver and blood after toxicant exposures

**DOI:** 10.21203/rs.3.rs-7610132/v1

**Published:** 2025-10-10

**Authors:** Bo Zhang, Benpeng Miao, Shuhua Fu, Wanqing Shao, Cristian Coarfa, Ravindra Kumar1, Prashant Kuntala, Bongsoo Park, Sandra Grimm, Rahul Jangid, Laurie Svoboda, Xiaoyun Xing, Daofeng Li, Shaopeng Liu, Robert Hamanaka, Claudia Lalancette, Maureen Sartor, Christopher Krapp, Gregory Crawford, Heather Patisaul, Tim Wiltshire, Shyam Biswal, Gokhan Mutlu, Sanjay Rajagopalan, Wan-Yee Tang, Marisa Bartolomei, Cheryl Walker, Dana Dolinoy, Justin Colacino, David Aylor, Ting Wang

**Affiliations:** Washington University School of Med; Washington University School of Medicine in St. Louis; Washington University School of Medicine; Washington University School of Medicine; Baylor College of Medicine; Washington University School of Medicine; Washington University School of Medicine in St. Louis; Johns Hopkins University; Baylor College of Medicine; Baylor College of Medicine; University of Michigan; Washington University School of Medicine; Washington University School of Medicine; Washington University School of Med; The University of Chicago; University of Michigan School of Public Health; University of Michigan School of Public Health; University of Pennsylvania; Duke University, Durham; North Carolina State University; University of North Carolina at Chapel Hill; Johns Hopkins University; University of Chicago; Case Western Reserve University; University of Pittsburgh School of Public Health; University of Pennsylvania; Baylor College of Medicine; University of Michigan School of Public Health; University of Michigan School of Public Health; North Carolina State University; Washington University in St. Louis

**Keywords:** Environmental epigenetics, surrogate and target tissue, transcriptome, epigenetic biomarkers, TaRGET II Consortium, early-life exposure

## Abstract

Exposure to toxic substances, particularly early in life, can perturb epigenomic marks linked to disease susceptibility. Human studies of environmental exposures often rely on surrogate tissues such as blood, but toxicant accumulation differs across organs and results in tissue-specific responses. Thus, understanding whether exposure-induced epigenomic alterations in surrogate tissues such as blood reflect changes in toxicant target tissues, such as liver, is essential for designing and interpreting environmental epigenetic studies. To address this knowledge gap, we systematically analyzed 1,013 multi-omics data from the TaRGET II Consortium, comparing molecular responses in mouse liver and blood following perinatal exposure to arsenic, lead, bisphenol A, tributyltin, di-2-ethylhexyl phthalate, tetrachlorodibenzo-p-dioxin, or air pollution in the form of particulate matter < 2.5μm (PM2.5). Most toxicant-induced molecular changes were tissue-specific, yet we identified a subset of co-regulated genes and regulatory elements in liver and blood in response to early-life exposure to toxicants. Moreover, we discovered that specific pathways, such as immune-related processes, were commonly affected by exposures in both tissues, and transcription factors, including *Klf*, *Jun*, *Ets1*, and *Cebp*, emerged as shared regulators. While molecular alterations are infrequently conserved between tissues following toxicant exposure, the shared alterations in transcription factors and biological pathways may provide a strategy to link effects in surrogate tissues to target tissues.

## Introduction

Exposure to environmental toxicants poses significant risks to human health, particularly when exposures occur early in life^1–4^. Developmental processes *in utero* and early after birth require complex and well-organized regulatory mechanisms that orchestrate the formation of mature organs and tissues from a single fertilized embryo^5,6^. Central to these mechanisms are epigenetic modifications, mitotically heritable marks that regulate gene expression and ultimately cell and tissue identity. Environmental exposures during development can dysregulate epigenetic programming processes, leaving an epigenetic “fingerprint” that may persist for years after exposure^1,7–9^. These “fingerprints” could differ based on the mechanism of action of the exposure. Environment-induced epigenetic alterations could be biomarkers of exposure or mechanistic drivers of disease processes later in life^10^. Developing such biomarkers could be useful for assessing toxicant exposures in populations, potentially months or years afterwards^11^. Epigenetic biomarkers that are correlated with disease incidence will be both valuable clinically and help resolve long-standing research questions about the developmental origins and epigenetic etiologies of disease.

Since early-life exposures may impact epigenetic programming and differentiation processes during embryogenesis, it is possible that these exposure-associated “fingerprints” could be a persistent signature of exposure detectable across multiple tissues. For example, metastable epialleles have been described as a rare class of epigenetic marks that are variable across individuals but have a high correlation in epigenetic state within an individual across tissues derived from the three germ layers (ectoderm, endoderm, mesoderm)^12–14^. Imprinted genes, which are expressed only from the maternal or paternal allele and the other allele epigenetically silenced, are another class of genes with tight epigenetic regulation that are also modifiable by the environment^15–17^. Thus, there is the potential that environmental exposures early in life may cause reprogramming at these sites, leaving a persistent and detectable signature of exposure in many different tissues. Understanding the consistency of these epigenetic signatures across tissues is particularly salient given that in human epigenetic epidemiology studies, epigenetic alterations are often measured only in an easily accessible “surrogate” tissue like blood. While these studies often assume that signatures detected in surrogate tissues reflect those in disease-relevant “target” tissues such as the liver or the brain, this assumption has not been thoroughly tested. Here, we present results from a study designed specifically to test the correspondence of environmentally induced epigenetic fingerprints across multiple tissues for a range of chemical toxicants.

The Toxicant Exposures and Responses by Genomic and Epigenomic Regulators of Transcription II (TaRGET II) consortium (T2C) was established by the National Institute of Environmental Health Sciences (NIEHS) to understand how the epigenome responds to early-life environmental exposures and evaluate the consistency of environmentally-induced epigenetic signatures between “surrogate” and “target” tissues^18^. TaRGET II performed *in vivo* mouse model experimental interrogation of the molecular effects of exposure to a wide range of toxicant exposures, including the endocrine disrupting compounds bisphenol A (BPA), tributyltin (TBT), diethylhexylphthalate (DEHP), and 2,3,7,8-tetrachlorodibenzo-p-dioxin (TCDD), along with metals and metalloids lead (Pb) and Arsenic (As), and particulate matter less than 2.5 microns in diameter (PM2.5). All exposures began 2 weeks prior to conception and carried through to offspring weaning at three weeks of age, at which point exposures were ceased and offspring were followed into adulthood to monitor the persistence and consistency of early life exposure-mediated environmental changes to the epigenome across exposures and tissues.

In this study, we leverage the comprehensive multiomics dataset generated by the TaRGET II consortium to explore transcriptomic and epigenomic changes in mouse “surrogate” blood, a mesoderm-derived tissue, and “target” liver, an endoderm-derived tissue, under early-life exposure to the same environmental toxicants. We systematically identified gene expression changes, chromatin accessibility alterations, and DNA methylation changes in blood and liver tissues from both male and female animals. Our findings reveal that the vast majority of molecular changes in response to a specific toxicant were tissue-specific. However, a small set of genes and regulatory elements was co-regulated between the two tissues, highlighting potential biomarkers for monitoring toxicant exposure in both the target liver and surrogate blood tissues. Moreover, we discovered that specific pathways, particularly immune-related pathways, were commonly altered in both liver and blood following exposure to distinct environmental toxicants. Additionally, we found that certain transcription factors could serve as common regulators in both liver and blood in response to environmental toxicant exposure. These insights may provide valuable guidance for the development of reliable biomarkers and help elucidate the molecular mechanisms underlying the tissue-specific and systemic effects of environmental toxicants.

## Results

### Early-life exposures to environmental toxicants induce significant transcriptomic and epigenetic remodeling in surrogate blood tissue

**Figure 1:**
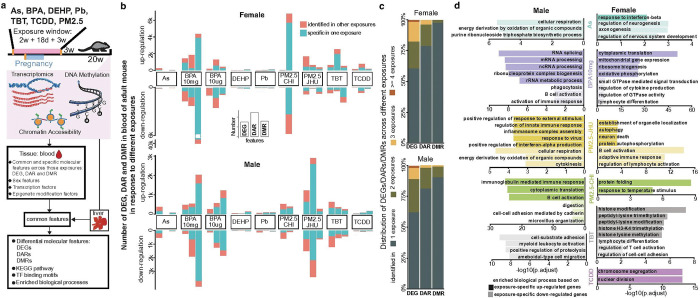
Early-life environmental exposures induce molecular changes in blood. **a)** Schematic overview of the study workflow. **b)** Numbers of up- and down-regulated differentially expressed genes (DEGs), differentially accessible regions (DARs), and differentially methylated regions (DMRs) in blood, shown separately for females and males across different exposures. Red bars indicate molecular features that were shared across multiple exposures, while green bars represent features specific to a single exposure. **c)** Distribution of molecular features across exposures. Colors indicate the percentage of DEGs/DARs/DMRs found in one exposure, two exposures, three exposures, or more than three exposures, separately for each sex. The majority of molecular features were unique to a single exposure. **d)**Gene Ontology (GO) terms enriched in upregulated and downregulated DEGs across exposures, presented separately for females and males. Dark-colored bars represent GO terms enriched among upregulated genes, while light-colored bars represent those enriched among downregulated genes.

The TaRGET II consortium was established to systematically detect transcriptional and epigenetic changes in response to early-life exposures to a wide range of environmental toxicants, including arsenic (As), lead (Pb), bisphenol A (BPA) at two doses, tributyltin (TBT), di-2-ethylhexyl phthalate (DEHP), the dioxin tetrachlorodibenzo-p-dioxin (TCDD), and particulate matter < 2.5μm (PM2.5) air pollution from two locations, Chicago (CHI) and Baltimore (JHU). This consortium profiled the transcriptome, chromatin accessibility, and DNA methylome of distinct target tissues (such as the liver and brain) and surrogate blood tissue at three different developmental stages. In this study, we focused on exploring the molecular changes and co-regulation of gene networks in target liver tissues and surrogate blood tissues. We exposed mice during embryonic development through the weanling stage (3 weeks old), then we analyzed RNA-seq, ATAC-seq, and WGBS data from 541 liver and 472 blood samples of early adult mice (5 months old) (Supplementary Data 1). For each exposure, the study included multiple replicates (n = 6 as designed) for both female and male mice to detect molecular changes in both sexes. This approach allowed us to explore the commonalities and tissue-specific responses of blood and liver to early-life exposures to environmental toxicants at different molecular levels (Fig. 1a).

We first identified potential blood biomarkers associated with early-life exposures by analyzing differentially expressed genes (DEGs), differentially accessible regions (DARs), and differentially methylated regions (DMRs) in surrogate blood tissue, compared to 5-month-old normal controls (Fig. 1b and Supplementary Datas 2–4). We observed distinct molecular changes across each of the exposures. Differences between exposures were seen in both the magnitudes of responses, as well as the identity of genes or epigenetic features. For example, we found 12,333 DARs in female mice exposed to PM2.5-CHI, but only 142 DARs in female mice exposed to DEHP. Pb, DEHP, and TCDD exposures induced relatively limited numbers of DEGs in both females and males compared to other exposures. We also saw little overlap between males and females in blood, an observation consistent across all tissues in TaRGET II data and described in detail elsewhere^19^. For example, exposure to 10 mg of BPA induced 3,105 DEGs in female blood, while only 347 DEGs were identified in male blood. PM2.5-JHU exposure induced 6,324 DARs in male blood but only 751 DARs in female blood. In contrast, high-dose BPA exposure (10 mg) strongly affected DNA methylation in both females and males. There was significant hypomethylation in female blood for this exposure with 46,478 DMRs, which dwarfed all other methylation responses by an order of magnitude (Fig. 1b).

All three assays revealed a signature for each exposure in blood. However, these varied widely in magnitude and there were no consistent patterns among the three molecular assays, either within or between exposures. For instance, some exposures had a strong open chromatin signature with little methylation signature, whereas the others had a strong methylation signature with few changes in open chromatin. In some cases, strong sex-specific effects were observed across assays. As an example, looking at gene expression in PM2.5-CHI exposed mice resulted in 1,130 DEGs in male blood, compared to only 61 in females. That same exposure altered chromatin accessibility at 12,488 DARs in females but induced only 501 DARs in males. Our findings highlight that molecular changes in surrogate blood tissue due to early-life exposure to environmental toxicants are highly sex-specific and exposure-specific, and the nature of epigenetic reprogramming (DNA methylation, chromatin accessibility) may vary as a function of the specific context.

We then explored blood biomarkers that were in common across different exposures. Although most biomarkers were exposure-specific, a substantial number (~ 40% of DEGs, ~ 20% of DARs, ~ 5% of DMRs) were shared across at least two exposures (Fig. 1c). Most shared DEGs were only shared by two or three exposures (Extended Data Fig. 1a, b). In females, the two different doses of BPA accounted for around half of the shared DEGs (701 down-regulated and 110 up-regulated). Interestingly, many of these common BPA-responding genes were also affected by PM2.5-JHU exposure but with a reversed up-regulated pattern (Extended Data Fig. 1a). In males, a set of genes (495) responding to PM2.5-JHU also showed similar expression changes under TBT exposure (Extended Data Fig. 1a). Gene ontology analysis of these common blood biomarkers indicated that many of them were highly enriched in immune-associated biological processes in both males and females, as expected (Extended Data Fig. 1b, c). Many immune-response genes, such as immunoglobulin (IgI, IGH, IGK) and killer cell lectin-like receptor (KLR), frequently exhibited changes in expression under multiple (n ≥ 4) early-life exposures (Extended Data Fig. 1c). This suggests that early-life exposure to environmental toxicants can impact immune function even 5 months after exposure.

We consistently found that each toxicant exposure left a specific signature. In other words, the signature associated with any exposure and sex (e.g., TBT-exposed female) could easily be distinguished from any other signature (Fig. 1b, Extended Data Fig. 1d). Gene ontology (GO) enrichment analysis suggested that biological processes and functions were also affected in an exposure-specific manner (Fig. 1d). In males, BPA 10mg exposure affected RNA splicing, while TBT exposure impacted cell-substrate adhesion. In females, protein translation and oxidative phosphorylation were affected by BPA 1mg exposure, and TCDD exposure disrupted cell division. We asked if any DARs or DMRs overlapped with Imprint Control Regions (ICRs), which have previously been reported as susceptible to early-life reprogramming^20,21^. We found 13 DARs that overlapped an ICRs and 75 DMRs that overlapped an ICR (Supplementary Data 5). We concluded that epigenetic signatures of past exposures were readily identifiable in blood, that these signatures could be captured by any of three molecular assays, that each chemical toxicant left a distinct signature, and that all signatures were sex-specific. Together, these findings highlight the complex and varied molecular consequences of different environmental toxicants, emphasizing the potential long-term impact of early-life exposures on the blood epigenome.

### Exposure-induced sex-specific transcriptomic and epigenetic remodeling in blood

**Figure 2:**
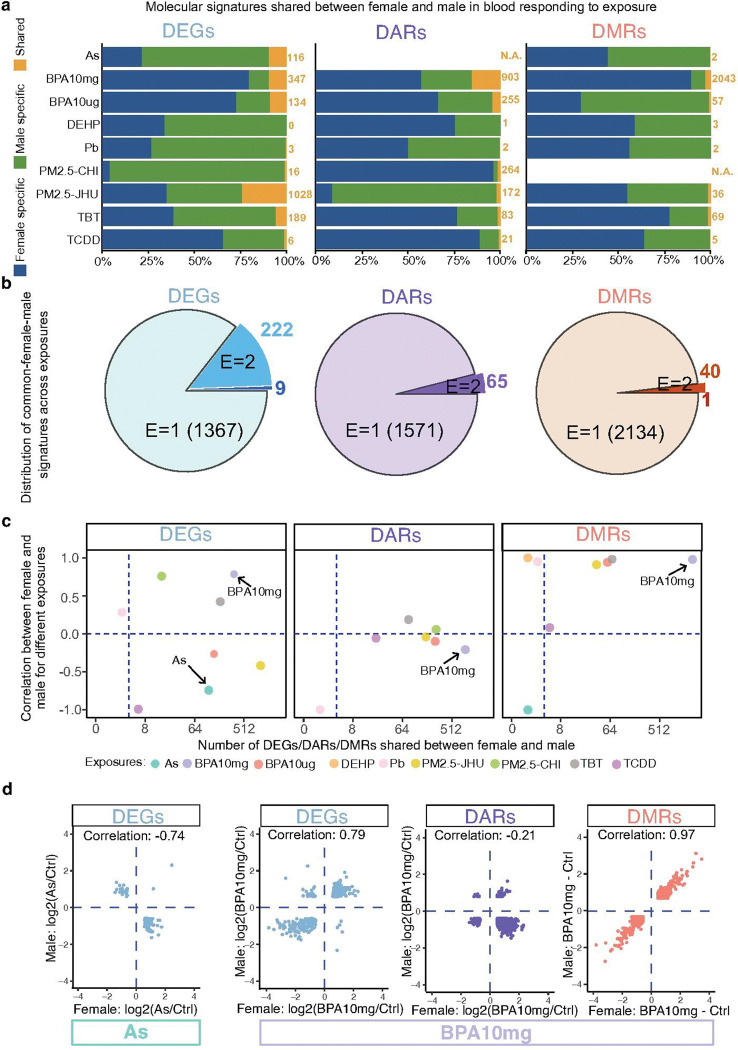
Sex-specific and shared molecular responses to environmental exposures in blood. **a)** Distribution of DEGs, differentially accessible regions (DARs), and differentially methylated regions (DMRs) between female and male samples for each exposure. Blue, green, and orange colors indicate features specific to females, specific to males, and shared between both sexes, respectively. A small proportion of DEGs, DARs, and DMRs were commonly regulated in both sexes across all exposures. **b)**Distribution of shared female–male molecular signatures across exposures. Colors represent DEGs, DARs, and DMRs shared between sexes that were identified in one (E=1), two (E=2), or more than two exposures. **c)** Pearson correlation between female and male responses for DEGs, DARs, and DMRs across exposures. The x-axis shows the number of shared molecular features, and the y-axis indicates the correlation coefficient between female and male fold changes. Each color represents a different exposure. **d)** Comparison of molecular responses between female and male to arsenic (As) (left panel) and BPA-10 mg/kg (right panel). In the As group, DEG expression changes were negatively correlated between sexes. In the BPA, both DEGs and DMRs showed positive correlations between females and males.

At both transcriptomic and epigenetic levels, the exposure-induced blood biomarkers were largely sex-specific, with only a small fraction of DEGs, DARs, and DMRs shared between females and males for each exposure. This was particularly evident in the methylation data (Fig. 2a and Supplementary Data 6). For the set of shared biomarkers between females and males, the majority of which were exposure-specific (Fig. 2b). Notably, only 9 common female-male DEGs were identified in more than three exposures (Extended Data Fig. 2a), and they often changed in opposite directions across sexes. For the shared DEGs, females and males showed very dynamic correlations when responding to exposures (Fig. 2c). Specifically, arsenic (As) and TCDD exposures induced strong reversed expression changes between the two sexes (Fig. 2d, Extended Data Fig. 2b). Notably, only 6 genes were commonly changed under TCDD exposure, and all of them exhibited reversed expression patterns (Extended Data Fig. 2b). In contrast, BPA10mg and PM2.5-CHI exposures induced strong similar expression changes between the sexes (Fig. 2d, Extended Data Fig. 2c).

Interestingly, unlike gene expression changes, the changes in chromatin accessibility for regions shared between the two sexes were not correlated between male and female mice, except for minor negative correlation seen with BPA10mg exposure (Fig. 2c and d). Conversely, DNA methylation changes were very consistent between male and female mice across multiple exposures, including BPA10mg (2,043 DMRs), BPA10μg (57 DMRs), PM2.5-JHU (36 DMRs), and TBT (69 DMRs) (Fig. 2c, d). These consistent DNA methylation alterations across sexes highlight the potential reliability of DNA methylation as a biomarker for detecting past exposure to various types of environmental toxicants.

### Toxicant exposure induced changes of transcription factors and EpiGenes in blood

**Figure 3:**
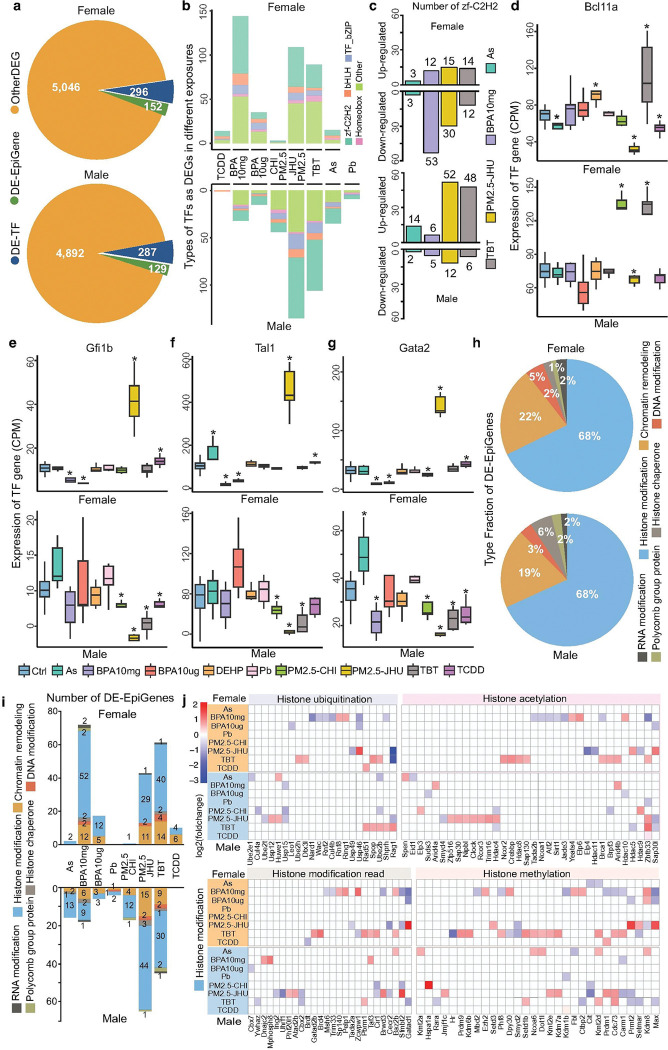
Environmental exposures cause different expression of transcription factors (DE-TFs) and epigenetic regulators (DE-EpiGenes). **a)** Total number of DEGs categorized as transcription factors (TFs) or epigenetic modification genes (EpiGenes) across exposures in females and males. DE-TFs and DE-EpiGenes make up about 10% of the union of DEGs in response to various exposures. **b)** Distribution of DE-TF types across exposures in females and males. The y-axis shows the number of DE-TFs, and colors represent different TF families. **c)** Number of upregulated and downregulated zf-C2H2-type DE-TFs across exposures in females and males. Each color indicates a different exposure, with most exposures downregulating zf-C2H2 TFs in females but upregulating them in males. **D-g)**Expression levels (CPM) of representative DE-TFs (Bcl11a, Gfi1b, Tal1, and Gata2) in control and exposed samples of females and males. Colors indicate sample groups. Asterisks (*) mark statistically significant differences between control and exposure groups (p < 0.05, Student’s t-test). **h)** Proportion of DE-EpiGenes in various epigenetic regulatory categories in females and males. Colors show different epigenetic functions. **i)**Number of DE-EpiGenes by category across exposures, shown separately for females and males. The y-axis shows gene counts, and colors denote regulatory types. **j)** Log2 fold-change in expression of DE-EpiGenes linked to histone modifications across exposures. Histone regulators are grouped into ubiquitination, acetylation, methylation, and histone mark readers. Red bars indicate upregulation; blue bars indicate downregulation.

Since exposure to environmental toxicants can trigger significant responses in peripheral blood, we further explored how transcription factor (TF) activity and epigenetic regulators (EpiGenes) were altered by early-life exposure to environmental toxicants. In both males and females, about 10% of exposure induced DEGs were TFs and EpiGenes, which responded to at least one exposure (Fig. 3a, Supplementary Datas 7 and 8). We found that PM2.5-JHU and TBT exposures induced a similar number of TF changes in both males and females. However, BPA10mg exposure induced a significantly higher number of changed TFs in female blood, while PM2.5-CHI exposure induced more TF changes in male blood (Fig. 3b).

In both females and males, nearly half of the differentially expressed TFs belonged to the zf-C2H2 type, containing the C2H2 zinc finger domain, which modulates important cellular processes such as proliferation, differentiation, and stress response^22,23^. In total, there were 120 zf-C2H2 differentially expressed TFs identified in females and 133 in males, with 53 TFs altered in both sexes (Extended Data Fig. 3). Clear sex-specific patterns were observed for these differentially expressed zf-C2H2 TFs. BPA10mg exposure significantly down-regulated zf-C2H2 TFs in females (53 down-regulated and 12 up-regulated), whereas only 11 zf-C2H2 TFs showed altered expression in males. PM2.5-JHU also induced more down-regulated (30) than up-regulated (15) zf-C2H2 TFs in female blood. Conversely, in male blood, PM2.5-JHU, TBT, and As exposures significantly upregulated the expression of zf-C2H2 TFs (52 in PM2.5-JHU, 48 in TBT, and 14 in As), compared to a limited number of down-regulated zf-C2H2 TFs (Fig. 3c). *Bcl11a*, an essential C2H2 transcription factor required for the switch from fetal hemoglobin (HbF) to adult hemoglobin (HbA) during development^24^ and for B lymphocyte differentiation^25^, was significantly upregulated in blood under PM2.5-CHI (male) and TBT (male and female) exposure. However, it was downregulated under PM2.5-JHU (female) exposure (Fig. 3d). *Gfi1b*, another crucial C2H2 transcription factor involved in blood cell development, particularly in erythropoiesis and megakaryopoiesis^26^, was significantly downregulated in blood under exposure to BPA (female), TBT (male), and PM2.5-JHU (male). Conversely, *Gfi1b* was significantly upregulated under PM2.5-JHU (female) exposure (Fig. 3e).

Other transcription factors, such as bHLH and GATA factors, also exhibited sex-specific changing patterns in response to environmental toxicant exposures. *Tal1*, a bHLH factor crucial for blood development and implicated in certain blood cancers like T-cell acute lymphoblastic leukemia (T-ALL)^27^, was downregulated under BPA10μg exposure in female blood but upregulated in male blood. Conversely, under PM2.5-JHU exposure, *Tal1* was upregulated in female blood but downregulated in male blood (Fig. 3f). *Gata2*, which plays a critical role in the early stages of blood cell formation, particularly in the development of hematopoietic stem cells^28^, also showed sex-specific expression changes. Under PM2.5-JHU exposure, Gata2 was upregulated in female blood but downregulated in male blood. In contrast, under BPA10mg exposure, *Gata2* was downregulated in both sexes (Fig. 3g). These observations underscore the sex-specific nature of transcriptional responses to environmental toxicant exposures. The differential regulation of key transcription factors highlights the complex interplay between sex and environmental factors in modulating gene expression, which may have significant implications for blood cell development and disease susceptibility.

Furthermore, we investigated differentially expressed epigenetic regulators (EpiGenes) in response to each exposure. In total, there were 152 and 129 EpiGenes in female and male with altered expression in response to at least one exposure, with 90% and 87% of them directly associated with histone modifications and remodeling (Fig. 3h). In females, BPA10mg, PM2.5-JHU, and TBT exposures induced the most changes in EpiGenes. However, some exposures, such as As, PM2.5-CHI, and Pb, induced almost no changes in EpiGenes. In males, PM2.5-JHU and TBT exposures led to the strongest changes in EpiGenes. In contrast, TCDD exposure did not induce any changes in EpiGenes, and Pb exposure only induced changes of 3 EpiGenes (Fig. 3i).

We then classified the EpiGenes based on their roles in histone modification and divided them into groups related to histone modification reading, histone ubiquitination, acetylation, and methylation. For each functional category, we observed strong sex-specific patterns (Fig. 3j).

TBT exposure induced the upregulation of four (in females) and five (in males) genes associated with histone ubiquitination. For instance, *Rag1*, which can ubiquitinate histone H3 and plays crucial roles in V(D)J recombination during immune cell differentiation and maturation^29,30^, was upregulated in TBT-exposed male blood but downregulated in female blood. TBT exposure also led to the upregulation of 8 histone acetylation genes in females, while distinct histone acetylation genes (3) were downregulated in males. This indicates that TBT can induce sex-specific histone acetylation reprogramming. Some histone methylation-related genes showed similar expression changes in both sexes. For example, *Carm1* was downregulated in both males and females under BPA10mg exposure. Additionally, *Cdc73*, *Kmt2d*, and *Setd1b* were all upregulated in both sexes under TBT exposure (Fig. 3j). These findings highlight the significant impact of environmental toxicants on epigenetic regulation, specifically through histone modifications, in a sex-specific manner. The differential regulation of genes involved in histone ubiquitination, acetylation, and methylation suggests that early-life exposure to toxicants might lead to complex, yet distinct, epigenetic reprogramming in males and females.

### The common molecular signatures between blood and liver in responding to environmental exposures

Having already established epigenetic biomarkers of exposure in blood, we compared exposure-induced molecular changes in blood and liver. Specifically, we looked for shared influences of each environmental exposure on both tissues. This analysis informed our larger objective of demonstrating how blood, an easy-to-sample surrogate tissue, could provide information on the epigenetic state of other tissues that are relevant to environmentally influenced diseases.

**Figure 4:**
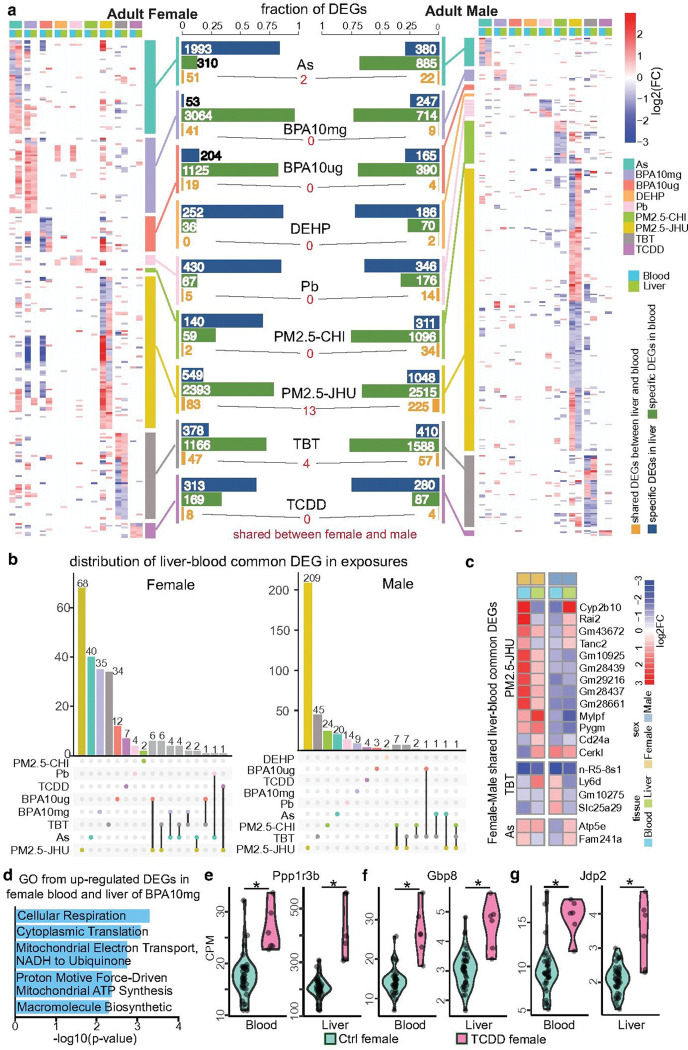
Shared differentially expressed genes (DEGs) between blood and liver in response to environmental exposures. **a)** Number and overlap of blood-liver common DEGs identified in females and males across different exposures. The bar plot shows the proportion of DEGs specific to liver (blue), specific to blood (green), and shared between blood and liver (orange) for each exposure in females (left) and in males (right). The accompanying heatmaps display the log_2_fold change of these shared DEGs in liver and blood across exposures in females (left) and in males (right). The red number in the middle indicates the number of blood-liver common DEGs in females that were also shared by males. **b)** Distribution of blood-liver common DEGs across exposures in females and males. The y-axis shows the number of shared DEGs that were either unique to one exposure or common to multiple exposures. **c)** Blood-liver common DEGs shared between both sexes. Red and blue bars indicate upregulated and downregulated genes, respectively. d) GO terms enriched among upregulated blood-liver common DEGs in females exposed to 10 mg/kg BPA. **e–g)** Upregulated gene expression of representative blood-liver common DEGs: Ppp1r3b, Gbp8, and Jdp2 in control and TCDD-exposed samples, shown for both blood and liver. Asterisks (*) indicate statistically significant expression differences (p < 0.05, Student’s t-test).

Robust molecular signatures in the liver for each exposure were identified and reported by the T2C in a separate publication^19^. Generally, molecular changes induced by each exposure showed strong tissue specificity. Only a very limited number of exposure-induced molecular signatures simultaneously affected both blood and liver at the transcriptome level (liver-blood common DEGs, Fig. 4a and Supplementary Data 9) or epigenetic level (liver-blood common DARs and DMRs, Extended Data Fig. 4 and Supplementary Datas 10–11). Consistent with the overall results in blood, the subset of liver-blood common DEGs were nearly all exposure-specific (Fig. 4b). No liver-blood common DEGs were found between the two doses of BPA exposure, but we identified 7 shared genes between two PM2.5 exposures in males. Similarly, there was almost no overlap between sexes. There were only 13 genes shared between females and males, including 6 non-coding genes and 7 protein-coding genes, with 7 genes being co-upregulated in female surrogate and target tissues but co-downregulated in males (Fig. 4c).

Exposure effects of gene expression exemplified the trend. On average, 1.83% of total DEGs found in either liver or blood were present in both tissues. There was at least some overlap between tissues for every exposure group except for DEHP-female. The PM2.5-JHU exposure induced a substantial number of DEGs in both tissues and in both sexes, resulting in the largest number of shared DEGs both in females (83) and in males (225). In addition, a moderate proportion (38%) of DEGs were regulated in opposite directions between tissues, placing the overall amount of co-regulation (same gene and in the same direction) somewhat less.

The proportion of blood DEGs that were also liver DEGs ranged from 0–14%. We must consider these data in light of the overall magnitude of exposure effects on gene expression. The overall number of DEGs in liver ranged from 94–2044, and the number of DEGs in blood ranged from 36–3105. We used a hypergeometric exact test to calculate the probabilities of seeing DEGs shared in both blood and liver (Supplementary Data 12), and found that four of twenty exposure groups displayed more sharing than would be expected by chance (*p* ≤ 0.05), and another three showed slight enrichment (*p* ≤ 0.1).

We used gene set enrichment analysis to investigate biological significance of the shared gene sets. The BPA10mg-female exposure group showed substantial co-regulation – 37 of the 41 liver-blood common DEGs were upregulated in both tissues. These genes were enriched in cellular respiration and mitochondrial electron transport processes (Fig. 4d), suggesting that high-dose BPA exposure strongly impacts energy metabolism in both target and surrogate tissues. Similarly, 7 of the 8 liver-blood common DEGs induced by TCDD exposure were co-regulated in females (Fig. 4a). This includes important genes such as *Ppp1r3b* and *Jdp2* (Fig. 4e-g), both of which have been previously reported to be dysregulated by TCDD exposure^31,32^. *Ppp1r3b* plays a crucial role in liver glycogen metabolism and blood glucose regulation, acting as a “metabolic switch” that determines how the liver stores energy^33^. *Gbp8* is involved in immune responses, liver inflammation, and infection^34^. *Jdp2* is associated with both liver cancer and bone homeostasis, and is involved in cell differentiation and chromatin remodeling^35–37^. Given their roles and their co-regulation by TCDD, *Ppp1r3b* and *Jdp2* have high potential to serve as biomarkers in blood to measure exposure to TCDD in target liver tissue.

At the epigenetic level, we noticed a distinct pattern for liver-blood common DARs (Differentially Accessible Regions) and DMRs (Differentially Methylated Regions). Similar to the gene expression data, the strong tissue-specific pattern of exposure-specific epigenetic changes also existed in liver and blood, with only a limited number of DARs and DMRs commonly affected by the same exposure in both surrogate and target tissues (Extended Data Fig. 4b and d). This may be largely due to the vast number of cis-regulatory elements in the genome. For example, 287 and 71 liver-blood common DARs were identified in males and females, respectively, after exposure to BPA10mg, with 93% of male common DARs showing decreased accessibility in response to BPA10mg. Conversely, 73% of female common DARs exhibited increased accessibility. In response to PM2.5-CHI, 155 liver-blood common DARs were identified, and 89% of them were co-regulated in both liver and blood.

The number of liver-blood common DMRs was even fewer. However, most of these common DMRs showed similar methylation changes in both surrogate and target tissues, except for female DMRs induced by TBT and male DMRs induced by BPA10mg. For PM2.5-JHU exposure, 13 out of 17 male common DMRs and 15 out of 18 female common DMRs were co-hypermethylated or co-hypomethylated. Additionally, all 9 common DMRs induced by DEHP and all 13 common DMRs induced by TCDD exhibited consistent changes in both liver and blood.

Overall, we conclude that a small component of the molecular responses to toxicants was shared across the two tissue types. We observed more concordant changes, meaning changes in the same direction, in the chromatin accessibility and DNA methylation data than in the gene expression data. This suggests that epigenetic biomarkers could be more reliable for measuring historical exposure to environmental toxicants.

### Common KEGG pathways in blood and liver responding to environmental exposures

With the identified liver-blood common molecular changes described above, we further explored the high-level commonality between blood and liver in response to environmental toxicants. We performed enrichment analysis of KEGG pathways for each exposure, based on DEGs from blood and liver (see Methods), and further examined whether any KEGG pathways were simultaneously impacted in both liver and blood by the same environmental toxicant exposure. These concurrent impacts indicate underlying biological processes that are commonly perturbed across tissues, potentially reflecting systemic effects of the environmental toxicants.

**Figure 5:**
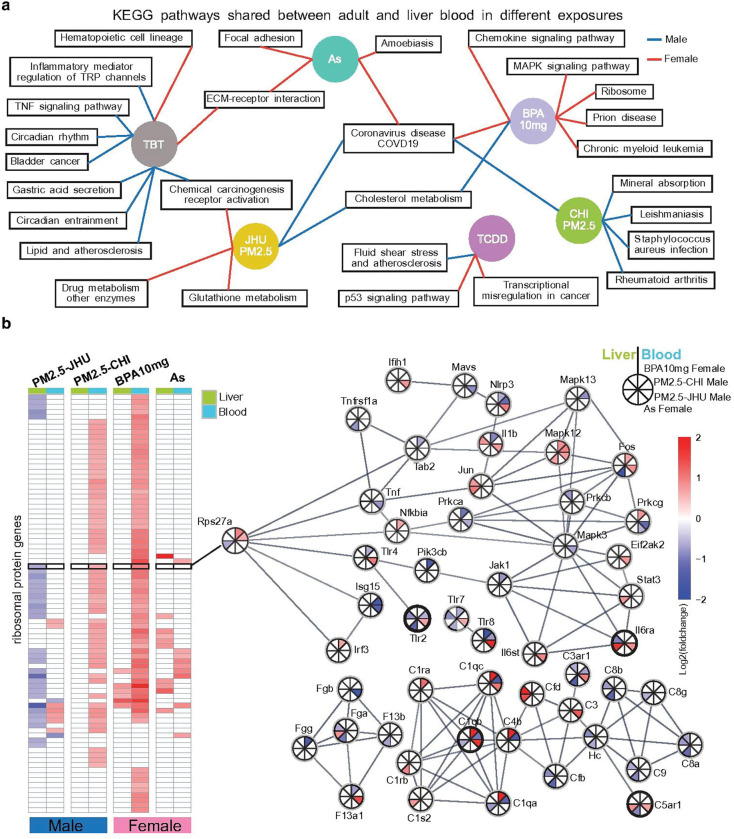
Environmental exposures induce shared KEGG pathway alterations in both blood and liver. **a)**KEGG pathways enriched by exposure-responsive DEGs in both blood and liver across multiple exposures. Each circle represents a KEGG pathway enriched in either tissue, with colors indicating different exposures. Squares mark KEGG pathways enriched in both blood and liver. Red and blue lines trace KEGG pathways identified in female and male samples, respectively. **b)** Shared enrichment of the Coronavirus disease–COVID-19 KEGG pathway in both blood and liver, observed across four distinct exposures. The left heatmap displays the log_2_fold-change of DEGs encoding ribosomal proteins in these exposures. The right panel shows the protein–protein interaction (PPI) network of non-ribosomal DEGs within the same KEGG pathway. Red and blue denote up- and down-regulated DEGs, respectively. Each row in the heatmap and each node in the network corresponds to a DEG. In the network diagram, the left half of each circle represents gene expression in the liver, and the right half represents blood, in response to exposure to BPA10mg in females, PM2.5-CHI in males, PM2.5-JHU in males, and As in females (top-down order).

Among all the exposures, we noticed that TBT perturbed the most common KEGG pathways between liver and blood in male animals, including inflammatory and TNF signaling, circadian rhythm, lipid metabolism, and chemical carcinogenesis (Fig. 5a and Supplementary Data 13). In females, TBT shared the ECM-receptor interaction pathway with As-exposed females. Notably, As exposure only perturbed common KEGG pathways in female animals, but not in males. Conversely, PM2.5-CHI exposure only perturbed common KEGG pathways in male animals, including those related to immunological response and mineral absorption (Fig. 5a). Interestingly, although BPA exposure is generally believed to mainly affect male animals, we found that most of the perturbed common KEGG pathways were in females, including those related to protein translation and folding, and MAPK signaling. There were limited blood-liver common KEGG pathways perturbed by multiple exposures. Significantly, the Coronavirus disease (COVID-19) related pathway was enriched in male animals under two PM2.5 exposures and in female animals under BPA10mg and As exposures.

We further explored the expression changes of genes belonging to the Coronavirus disease (COVID-19) related pathway. This pathway contains two sets of genes related to protein translation (ribosomal proteins) and immunological response. We observed strong tissue-specific gene dysregulation in liver and blood, which contributes to significant perturbation of this pathway by distinct gene combinations. In PM2.5-CHI-exposed males and BPA10mg-exposed females, a large set of ribosomal protein genes were upregulated in blood, while fewer genes were dysregulated in liver. In PM2.5-JHU-exposed males, 32 ribosomal protein genes were downregulated in liver tissue. As exposure induced the upregulation of ribosomal protein genes in both liver and blood (Fig. 5b and Supplementary Data 14).

For the immunological response gene set, most genes were dysregulated in a tissue-specific and exposure-specific fashion. However, several genes were affected by exposure in both liver and blood. *Tlr2*, which encodes Toll-like receptor 2 and plays a role in liver health, particularly in the context of inflammation and disease^38^, was downregulated in both liver and blood under PM2.5-CHI exposure. *Il6ra*, the alpha subunit of the Interleukin-6 receptor, crucial for liver function and regeneration^39^, was upregulated in both liver and blood under PM2.5-JHU exposure. *C5aR1* (complement component 5a receptor 1), which is involved in liver inflammation and fibrosis in the context of nonalcoholic steatohepatitis (NASH)^40^, was also upregulated in both liver and blood under PM2.5-JHU exposure. Meanwhile, several subunits of the complement component C1, such as *C1ra* and *C1qa/b/c*, were upregulated in female blood under BPA10mg exposure. These findings illustrate that the simultaneous impact on both protein translation and immunological response genes in blood and liver highlights the systemic effects of these toxicants and provides insights into the molecular mechanisms underlying their influence.

### Common transcription factor binding motifs harbored in exposure-DARs of blood and liver

Since we observed more concordant changes at the epigenetic level compared to gene expression changes, we explored the enrichment of transcription factor binding motif sites (TFBSs) harbored in the exposure-induced differentially accessible regions (DARs) in both liver and blood. We performed de novo motif finding in the more and less accessible DARs of liver and blood, and noticed a strong tissue-specific enrichment pattern of TFBSs for the same exposure (Extended Data Fig. 5 and Supplementary Data 15).

**Figure 6:**
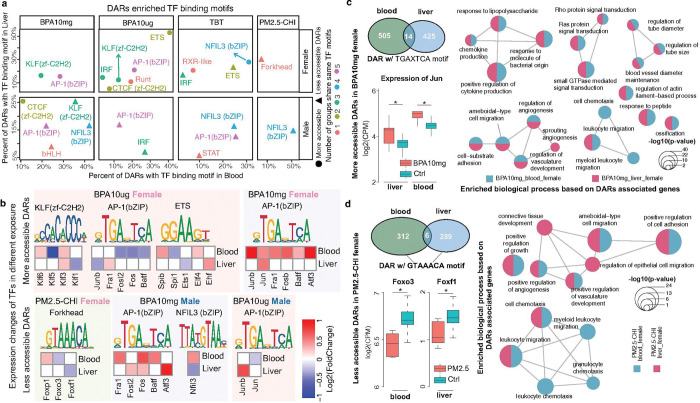
Transcription factor (TF) binding motifs enriched in differentially accessible regions (DARs) shared between blood and liver in response to environmental exposures. **a)** TF binding motifs enriched in DARs from blood and liver samples across exposures in females and males. The x-axis represents the percentage of DARs in blood containing specific TF motifs; the y-axis shows the corresponding percentage in the liver. Dots and triangles indicate motifs enriched in more accessible and less accessible DARs, respectively. Colors represent the number of exposure groups sharing the same blood–liver common TF binding motif. The Fra1 binding motif was identified as a shared feature in five exposure groups. **b)** Expression changes of TFs predicted to bind shared blood–liver DAR-enriched motifs. TF motif logos are shown, along with the corresponding TF genes expressed in blood and liver under different exposures. Cell colors represent log_2_(fold-change) of TF expression: red for upregulation and blue for downregulation. **c)** Blood-liver shared TF motif TGAXTCA is enriched in more accessible DARs from BPA-10 mg/kg exposed females. The top left panel shows the overlap of DARs containing the TGAXTCA motif in blood and liver. The bottom left panel shows the expression of Jun, a TF that binds this motif, in control and BPA-exposed samples. BPA-10 mg/kg induced upregulation of Jun in both tissues. The right panel displays enriched biological processes based on genes associated with more accessible DARs in the blood and liver. Red and blue circles indicate GO terms enriched in liver and blood, respectively. Circle size corresponds to the statistical significance (−log_10_p-value). **d)** Blood-liver shared TF motif GTAAACA, enriched in less accessible DARs in PM2.5-CHI-exposed females. The top left panel shows the overlap of DARs containing this motif in blood and liver. The bottom left panel presents the expression of TFs Foxo3 and Foxf1, both predicted to bind this motif, in blood and liver, respectively. PM2.5-CHI exposure led to downregulation of Foxo3 in blood and Foxf1 in liver. The right panel shows enriched GO terms from genes associated with less accessible DARs in both tissues, with color and size conventions as in panel (**c**).

However, certain TF families showed strong enrichment in both females and males across multiple exposures. Specifically, the AP-1 (Activator Protein-1), bZIP, and KLF families frequently appeared under multiple exposure conditions. The AP-1 family, which includes subfamilies like Jun, Fos, and ATF, plays crucial roles in regulating gene expression in response to various stimuli^41^. AP-1 motifs were enriched in the liver and blood of both females and males exposed to two doses of BPA (Fig. 6a). In females, AP-1 TFBSs were enriched in more accessible DARs induced by BPA exposure, suggesting increased binding opportunities for AP-1 factors. Conversely, in males, these TFBSs were enriched in less accessible DARs under the same exposure, indicating reduced binding opportunities. Such sex-specific patterns were also observed for other TF families: KLF motifs were enriched in the response to two doses of BPA exposure; bZIP motifs showed enrichment patterns in response to TBT exposure (Fig. 6a and Supplementary Data 16). These findings highlight the complex and sex-specific nature of transcription factor binding site enrichment in response to environmental toxicant exposures across target and surrogate tissues.

To determine the specific transcription factors (TFs) within each TF family, we calculated expression changes of TFs in each family with distinct exposure conditions in both liver and blood tissue (Methods) (Fig. 6b). In BPA10mg-exposed females, Jun of the AP-1 family was significantly upregulated in both blood and liver (Fig. 6b, c). We further explored the Jun/AP-1 motif (TGAXTCA) in more accessible DARs. There were a total of 519 and 439 DARs in blood and liver, respectively, containing the TGAXTCA motif, but only 14 DARs were simultaneously induced by BPA10mg in both blood and liver (Fig. 6c). We then investigated the function of genes around blood DARs and liver DARs. Many shared biological processes were identified in both sexes, including immune response, angiogenesis, and Rho protein signaling (Fig. 6c). Combined with the upregulated expression of Jun in both tissues, these results suggest that Jun could be the common master transcription factor (TF) in responding to BPA10mg exposure in females. Under PM2.5-CHI exposure, we identified the enrichment of the Forkhead family and NEIL3 (bZIP) family in the less accessible DARs in females and males, respectively. Similar to the AP-1 motifs in BPA10mg-induced DARs, we found limited overlap between liver and blood DARs with the FOX motif (GTAAACA). There were a total of 318 and 295 DARs in blood and liver containing the GTAAACA motif, but only 6 DARs were simultaneously induced by PM2.5-CHI in both blood and liver (Fig. 6d). Between blood and liver, we noticed that Foxo3 was downregulated in blood and Foxf1 was downregulated in liver (Fig. 6b, d). This suggests that distinct TFs from the same TF family respond to the same early-life exposure and affect similar biological processes, such as cell migration and angiogenesis, and leukocyte migration and activation, in both tissues (Fig. 6d).

## Discussion

Environmental toxicants are common in modern life, deriving from industrial activities, consumer products, and urban pollution^2–4^. Exposures to environmental pollutants, particularly early in life, can reprogram the epigenome, with detectable signatures throughout the life course^2–4^. Here, we tested the hypothesis that a perinatal exposure to endocrine disrupting toxicants, toxic metals and metalloids, and air pollution would lead to long-term epigenetic signatures in target and surrogate tissues derived from different germ layers, in this case blood (mesoderm) and liver (endoderm), and we proposed that some elements of these signatures would be concordant across the two tissues. In both tissues, we found distinct alterations across gene expression, DNA methylation, and chromatin accessibility several months after exposure. We found that these molecular signatures were overwhelmingly exposure-specific, sex-specific, and tissue-specific. There was little overlap in signatures in blood and liver for a given exposure. However, we did identify a small number of concordant sites as well as commonalities in biological processes altered by exposures across tissues, with consistency in altered predicted transcription factor binding in a subset of exposures.

In blood, we identified significant transcriptomic changes and dynamic epigenetic remodeling 5 months after exposure, indicating the considerable long-term impact of early-life exposure to environmental toxicants. These extensive molecular signatures, including changes in gene expression levels, chromatin accessibility, and DNA methylation, can serve as biomarkers to estimate previous exposure conditions. The ability to detect these molecular alterations highlights the potential for developing retrospective methods to assess the historical exposure to specific environmental toxicants. Importantly, these signatures were highly exposure-specific, suggesting that they depended on the chemical nature of the toxicant. The concordance between males and females varied by molecular assay. Gene expression changes between females and males had more common elements compared to epigenetic chromatin remodeling, but those changes were only positively correlated between sexes for a subset of chemicals (e.g., BPA10mg, TBT, PM2.5-CHI). Likewise, chromatin accessibility changes were mostly uncorrelated between sexes. However, DNA methylation changes showed strong positive correlations between females and males across most exposures. This high consistency of DNA methylation changes suggests the strong potential of DNA methylation to serve as reliable biomarkers for detecting previous exposure to toxicants in both sexes (Fig. 2c, d). These consistent DNA methylation alterations underline their robustness and reliability as indicators for assessing historical exposure to various environmental toxicants, making them promising candidates for monitoring and precision environmental health strategies. Moreover, our analysis of molecular signatures of liver and blood tissues revealed strong responses to early-life environmental toxicant exposures in both tissues. However, that response was largely tissue-specific, and very few elements were shared. On average, 4.0% of the genes altered in blood were also altered in liver, and not all of these overlapping genes showed concordant regulation in the same direction. Chromatin accessibility and DNA methylation in blood showed even less overlap with the liver (< 2% on average). However, DNA methylation did show the highest proportion of concordant changes.

On one hand, these findings suggest that blood-based molecular signatures may serve as useful surrogates for global gene regulatory changes and epigenetic remodeling in less accessible target tissues, like the liver. There was no case in which we saw a strong response in the blood and did not see a strong response in the liver. Our results strongly support epigenetic biomarkers in blood as indicators of broad-based toxicant effects in multiple tissues. On the other hand, the observed changes in blood did little to inform the specific changes that occurred in the target tissue. At present, molecular profiles from blood should be interpreted as evidence of exposure, rather than providing predictive value for specific changes in the epigenome of other tissues. Accurate interpretation of blood-based biomarkers will require a deeper understanding of the molecular mechanisms driving tissue specificity, as well as careful validation in both experimental and translational contexts.

We observed some higher-order regulatory commonalities across blood and liver that may begin to elucidate some of those mechanisms. In some cases, exposure perturbed similar biological processes in both tissues. For instance, TBT exposure perturbed the greatest number of common KEGG pathways between liver and blood in male animals. These shared pathways include inflammatory and TNF signaling, circadian rhythm regulation, lipid metabolism, and chemical carcinogenesis, all of which are critical for maintaining tissue homeostasis and responding to stress. Interestingly, despite this pathway-level overlap, only a limited number of genes were co-regulated (i.e., showed consistent differential expression) in both tissues. This suggests that the same signaling pathways and biological processes can be disrupted across tissues by a common exposure, but through the dysregulation of distinct molecular components in a tissue-specific manner. Furthermore, when examining multiple exposures, we found that immune-related pathways, particularly those involved in inflammation and cytokine signaling, were recurrently affected across tissues and exposures. This pattern implies that environmental toxicants broadly activate inflammatory responses, which may serve as a common mechanistic driver of tissue damage and long-term dysfunction.

In our analysis of chromatin accessibility remodeling, the results suggest that distinct transcription factors (TFs) within the same TF family can recognize similar or conserved DNA binding motifs within differentially accessible regions (DARs). These regions were induced by the same early-life exposure and are often linked to the regulation of similar biological processes across both surrogate (blood) and target (liver) tissues. This finding highlights the functional redundancy or specialization among family members in mediating transcriptional responses to environmental cues. Moreover, certain TF families—such as AP-1, bZIP, and KLF—were consistently enriched in chromatin accessibility changes in response to multiple environmental exposures, regardless of tissue type. These TFs are known to regulate key cellular processes such as inflammation, metabolism, and cell differentiation^41–48^, and their repeated involvement across exposures suggests a conserved regulatory logic underlying the chromatin response to environmental toxicants.

These observations suggested the importance of exploring the “TF binding grammar”—that is, the combinatorial and context-dependent rules by which TFs interact with regulatory elements such as enhancers and promoters. Understanding how environmental exposures alter this regulatory grammar across tissues and developmental stages will be essential for elucidating the molecular mechanisms of toxicant-induced gene regulation and for identifying potential intervention points for future therapeutic strategies. Taken together, our study emphasized the importance of considering both gene-level specificity and pathway-level convergence in evaluating the long-term systemic effects of toxicant exposure. These approaches hold promise for enabling surrogate tissue analysis in the absence of overlapping gene sets. Moreover, targeting inflammation and repressing the immune dysregulation may represent a promising therapeutic strategy to mitigate the harmful consequences of early-life exposure to environmental toxicants.

The development of molecular biomarkers in blood can form an important tool for precision medicine and precision environmental health. The ideal signature would be exposure-specific, consistent among exposed individuals, and permanent throughout the life course. Our results demonstrate the first two parameters. Separate analysis from the T2C found that molecular signatures can change with age^49^, and this must be accounted for in biomarker development. It is well-established that many tissues including blood and liver show strong sex differences in gene regulation^50–55^, and our results suggest that separate biomarkers of exposure may need to be developed for males and females. Nonetheless, the extreme exposure-specificity in our results motivate further development. Our results are from a tightly controlled exposure paradigm in mice and in most cases at a single dose. Future work in this area would test if toxicant-specific signatures are robust to different windows of exposures and different doses. We must also consider chemical mixtures and combinatorial effects. For example, if a mouse was experimentally exposed to both TBT and TCDD, we would hope to detect both the TBT profile and the TCDD profile. Such simple experiments will provide the building blocks for understanding environmental epigenetics in mixtures. In the future, we could imagine retrospectively inferring complex exposomes from individual molecular profiles.

## Method

### Animals and Exposures

C57BL/6 (B6; Jackson Laboratory, Bar Harbor, ME) mice were used for all experiments, except for the lead (Pb) and phthalate (DEHP) exposure studies, which employed wild-type non-agouti (a/a) mice derived from a > 230-generation colony of viable yellow agouti (Avy) mice.

Mice were exposed to toxicants perinatally via maternal diet, drinking water, or air breathing, with the exposure window spanning from pre-conception through weaning. In brief, two weeks before mating, virgin female dams (6–8 weeks old) were randomly assigned to the exposure or control group. Detailed methods for the animal exposure and tissue collection were described in our previous study^19^.

### Raw sequence data and processing

A total of 541 liver and 472 blood genome-wide data sets were generated from 20-week-old mice exposed to pollutants during early development, including arsenic (As), lead (Pb), bisphenol A (BPA), tributyltin (TBT), di-2-ethylhexyl phthalate (DEHP), 2,3,7,8-tetrachlorodibenzo-p-dioxin (TCDD), and particulate matter less than 2.5 μm in diameter (PM2.5). These data sets included 345 RNA-seq data, 316 chromatin accessibility data, and 352 DNA methylation data (WGBS).

Raw fastq files of RNA-seq data were processed by Cutadapt^56^ (v1.16; --quality-cutoff = 15,10 --minimum-length = 36), FastQC^57^ (v0.11.7), and STAR^58^ (v2.5.4b; --quantMode TranscriptomeSAM --outWigType bedGraph --outWigNorm RPM) to do the trimming, generating QC report and mouse genome mapping (mm10) using our own built pipeline, the TaRGET-II-RNA-seq-pipeline (https://github.com/Zhang-lab/TaRGET-II-RNAseq-pipeline). Next, featureCounts (v1.5.1)^59^ was used to calculate the gene expressions across normal and exposure samples within the pipeline based on GENCODE vM20 gene annotation of mouse genome^60^.

ATAC-seq fastq files were processed by our own built pipeline based on AIAP^61^, the TaRGET-II-ATAC-seq-pipeline (https://github.com/Zhang-lab/TaRGET-II-ATACseq-pipeline) that integrated AIAP packages, including optimized QC reports and analysis pipeline with default parameters to generate the open chromatin regions (OCRs). Then, the consensus regions of OCRs across all exposure and control samples were generated with Index (https://github.com/Altius/Index) method used for the downstream analysis^62^. The ATAC-seq signals of consensus OCRs were calculated by using the intersectBed method of bedtools^63^.

The TaRGET-II-WGBS-pipeline (https://github.com/Zhang-lab/WGBS_analysis) was built by using the Cutadapt^56^ (v1.16; --quality-cutoff = 15,10 --minimum-length = 36) and Bismark^64^ (v0.19.0; --bowtie2 -X 1000 --score_min L,0,−0.6 -N 0 --multicore 2 -p 4) to do the trimming and mouse genome mapping. The pipeline also incorporated quality control, generated user-friendly files for computational analysis, and output genome browser tracks for data visualization.

The RNA-seq and ATAC-seq datasets were normalized to correct for batch effects arising from different data production centers by using the method described in the previous study^19^, and then the consortium-normalized read count data were used for downstream analysis.

### Differential analyses

The differentially expressed genes (DEGs) between control and exposure samples under different conditions were identified by using DESeq2^65^ (v1.34.0) as previous studies^66,67^. Consortium-normalized read count data were used to identify the DEGs between the exposed and control samples. Genes were considered significantly differentially expressed if they met the following stringent criteria: an adjusted p-value (Benjamini-Hochberg correction) less than 0.001 and an absolute log2 fold change greater than log2(1.5), equivalent to a 1.5-fold change in expression.

Differentially accessible regions (DARs) between control and exposure samples were identified using edgeR (v3.36.0)^68^, as in previous studies^69,70^. Consortium-normalized ATAC-seq reads were used to identify DARs, with significance determined by the following cutoffs: an absolute log2 fold change greater than log2(1.5) (a 1.5-fold change in accessibility) and a false discovery rate (FDR) less than 0.01.

Differential methylation regions (DMRs) between control and exposure samples were identified using a method adapted from previous studies^71,72^. Methylation levels were quantified using whole-genome bisulfite sequencing (WGBS) data. Briefly, raw WGBS reads were aligned to the reference genome using Bismark with default parameters, followed by deduplication to remove PCR artifacts. Methylation levels at individual CpG sites were calculated as the ratio of methylated reads to total reads (methylated + unmethylated) at each site, with a minimum coverage of 20 reads per site to ensure reliability. DMRs were identified through a genome sliding approach, as previous studies^72,73^: the genome was divided into sliding 200bp windows, the methylated and unmethylated reads counts in each window were calculated in both control and exposed samples, and a Chi-squared test was applied to identify DMRs. Regions were defined as differentially methylated if they exhibited a methylation level difference of at least 0.1 (a 10% absolute difference in methylation between control and exposed condition) and a Q-value (adjusted for multiple testing using the BH method) less than 0.1. Additionally, only regions with at least 2 CpG sites and a minimum average coverage of 20 reads per site were considered to enhance statistical power and reduce noise in the analysis, and the neighboring regions were merged to maximize the width of DMRs.

### The changes of molecular features in blood under different exposures

The ggplot2 package of R was used to generate bar plots to show number of DEGs, DARs and DMRs across exposures in blood^74,75^. The enriched biological processes in different exposures based on DEGs were generated by clusterProfiler package of R using the mouse annotation database (org.Mm.eg.db)^76^. The heatmap function and UpSetR package of R was used to visualize the distribution of DEGs identified in multiple exposures^77^. The intersectBed method of bedtools was used to identify the overlapped DMRs (at least 1 bp) between different exposures, or between female and male, as the common signatures. Next, the coefficient correlation between females and males in different exposures was separately calculated by using the cor function of R based on changes of DEGs, DARs, and DMRs under exposures in females and males. The transcription factors (TFs) and epigenetic modification factors (EpiGenes) were separately gathered from the AnimalTFDB3.0 and Epi-Modifiers databases^78,79^. The differentially expressed TFs (DE-TF) and EpiGenes (DE-EpiGenes) were identified and assigned into different types based on the two downloaded annotation files.

### The common features between blood and liver in response to different exposures

The common features between blood and liver were identified in different exposures separately for female and male. The DEGs simultaneously affected in both blood and liver (blood-liver common DEGs) under different exposures were identified by the gene symbols annotated by GENCODE vM20. The blood-liver common DARs in different exposures were identified if genomic coordinates of DARs were same. The blood-liver common DMRs were measured by genomic intersection of DMRs (at least 1bp overlap) using the intersectBed method of bedtools. Then, exposure distribution of blood-liver common DEGs, DARs and DMRs were measured with above strategy and visualized by UpSet package of R. The Enrichr method was applied to identify the biology processes enriched in up-regulated DEGs in both female blood and liver of BPA10mg^80^.

The enrichKEGG function of clusterProfiler package in R was used to identify the KEGG pathways enriched in different exposures (Qvalue < 0.1) separately for blood and liver, then, common KEGG pathways between blood and liver were found in different exposures. The DEGs under exposures belonged to KEGG pathways were extracted by AnnotationDbi::select function in the package. Then, those genes interaction networks from liver blood common KEGG pathways were built by using the string database^81^.

The transcription factors binding motifs (TFBS) enriched in more and less accessible DARs of exposures from blood and liver were separately analyzed by using findMotifsGenome.pl (−size given) of HOMER software (v4.11.1)^82^. The significantly enriched de novo binding motifs were identified with following criteria that calculated by HOMER: (1) at least 5% of accessible DARs in exposure contained the TFBS; (2) the match score of TFBS should be > 0.8; (3) P value of TFBS should be < 1e – 11. Then, known transcription factors genes (TFs) that could bind the enriched TFBS were extracted with the match score at least greater than 0.8 and then classes of those TFs were annotated by the files downloaded from AnimalTFDB3.0 database. The number of DARs enriched common TFBS between liver and blood was measured and visualized by jvenn method. The sequence logs of TFBS were collected from JASPAR database^83^. The DARs containing TFs binding motifs were extracted from the HOMER results, and then the genes around those DARs in different exposures were used to identify the enriched biology processes by enrichGO function of clusterProfiler package. The GO terms were visualized by emapplot of clusterProfiler.

## Supplementary Material

Supplementary Files

This is a list of supplementary files associated with this preprint. Click to download.

• ExtendedDataFig1.pdf

• ExtendedDataFig2.pdf

• ExtendedDataFig3.pdf

• ExtendedDataFig4.pdf

• ExtendedDataFig5.pdf

• InventoryofSupportingInformation.docx

• SupplementaryData1.samplesummary.xlsx

• SupplementaryData2.adultbloodDEG.txt

• SupplementaryData3.adultbloodDAR.txt

• SupplementaryData4.adultbloodDMR.txt

• SupplementaryData5.ICRsummary.xlsx

• SupplementaryData6.adultbloodDEGTFfamily.txt

• SupplementaryData7.adultbloodDEGEpiGene.txt

• SupplementaryData8.commonfeaturesbetweenfemaleandmale.txt

• SupplementaryData9.LiverBloodadultsharegenelogFC.xlsx

• SupplementaryData10.LiverBloodadultsharedDARlogFC.xlsx

• SupplementaryData11.LiverBloodadultsharedDMRlogFC.xlsx

• SupplementaryData12.surrogateoverlaphypergeometricresult.xlsx

• SupplementaryData13.KEGGPathwayAllQvalue0.1shareliverblood.txt

• SupplementaryData14.Log2FCGenefromCOVID19.txt

• SupplementaryData15.DARenrichedCommonmotifbetweenbloodandliver.txt

• SupplementaryData16.commonbloodandliverMotifgeneexp.txt

## Data Availability

RNA-seq, ATAC-seq and WGBS data in the paper has been deposited through Gene Expression Omnibus (GEO) repository: GSE146508 and those data are also available at the TaRGET II data portal (https://data.targetepigenomics.org/). All analysis results are visualized at accompanying database ToxiTaRGET: https://toxitarget.com/. Custom codes are available at GitHub: https://github.com/BenpengMiao/Surrogate_and_Target.
